# mTOR/α-ketoglutarate signaling: impact on brain cell homeostasis under ischemic conditions

**DOI:** 10.3389/fncel.2023.1132114

**Published:** 2023-05-11

**Authors:** Iryna Lushnikova, Olha Kostiuchenko, Magdalena Kowalczyk, Galyna Skibo

**Affiliations:** ^1^Department of Cytology, Bogomoletz Institute of Physiology, National Academy of Sciences of Ukraine, Kyiv, Ukraine; ^2^Institute of Biochemistry and Biophysics, Polish Academy of Sciences, Warsaw, Poland

**Keywords:** mTOR, α-ketoglutarate (αKG), homeostasis, brain ischemia, autophagy, neuroprotection

## Abstract

The multifunctional molecules mechanistic target of rapamycin (mTOR) and α-ketoglutarate (αKG) are crucial players in the regulatory mechanisms that maintain cell homeostasis in an ever-changing environment. Cerebral ischemia is associated primarily with oxygen-glucose deficiency (OGD) due to circulatory disorders. Upon exceeding a threshold of resistance to OGD, essential pathways of cellular metabolism can be disrupted, leading to damage of brain cells up to the loss of function and death. This mini-review focuses on the role of mTOR and αKG signaling in the metabolic homeostasis of brain cells under OGD conditions. Integral mechanisms concerning the relative cell resistance to OGD and the molecular basis of αKG-mediated neuroprotection are discussed. The study of molecular events associated with cerebral ischemia and endogenous neuroprotection is relevant for improving the effectiveness of therapeutic strategies.

## 1. Introduction

The brain is one of the most energy-consuming organs, so nutrient and oxygen deficiency is critical for cellular homeostasis. Complex signaling pathways and cooperation of molecular mechanisms ensure the homeostasis of brain cells, thus maintaining the viability and functioning of neurons with minor fluctuations in the extracellular environment. When the threshold of ischemic resistance is exceeded, a metabolic imbalance in the brain tissue occurs, followed by progressive neuronal degradation. Oxygen-glucose deficiency (OGD) initiates ischemic injury as a result of energy-limiting and dysregulated signaling pathways (Cespedes et al., 2019; Datta et al., 2020; Fakih et al., 2022). However, functional failures can be reversible at the primary stage, and pharmacological correction at the molecular level could either prevent a destructive course or promote the restoration of tissue status and brain functions (Rosarda et al., 2021; Qin et al., 2022). Based on this, a comprehensive study of the molecular mechanisms of cellular adaptation, neurodegeneration, and neuroprotection in experimental models of cerebral ischemia is appropriate.

Oxygen-glucose deficiency-induced changes in the composition of the microenvironment transform intracellular metabolism, including events that are modulated by mTOR (mechanistic/mammalian target of rapamycin). This multifunctional molecule is one of the base elements for maintaining cellular homeostasis, while dysregulation of mTOR signaling is associated with many cerebral diseases, including ischemic stroke (Wong, 2013; Saxton and Sabatini, 2017). It is reported that under ischemic conditions, activation of autophagy induced by mTOR-suppression has a protective effect on cell survival, whereas excessive autophagy causes apoptosis and/or necrosis of neurons (Hou et al., 2019; Zhang et al., 2021). Exogenous or endogenous regulation of mTOR metabolism is reasonable in terms of possible correction of neurodegeneration. Numerous studies discuss the versatility of the properties of αKG (α-ketoglutarate) and its regulatory role in maintaining cellular metabolic balance (Zdzisińska et al., 2017; Wilson and Matschinsky, 2021; Hansen and Gibson, 2022). αKG is not only an intermediate of the tricarboxylic acid (TCA) cycle but also is involved in amino acid metabolism, regulation of signaling pathways, and the scavenging of reactive oxygen species (ROS), thereby ensuring cellular homeostasis and survival in adverse environmental conditions (Liu et al., 2018). Signaling cooperation between αKG and mTOR is often discussed in the context of metabolic regulation. There is conflicting evidence on whether αKG suppresses or activates mTOR function, and how it contributes to the protective effects of αKG.

Due to their heterogeneous functions and the constant availability in cells, both mTOR and αKG largely determine metabolic homeostasis under OGD conditions. The main patterns of mTOR/αKG-mediated mechanisms are widely described (Harrison and Pierzynowski, 2008; Kim et al., 2017; Zdzisińska et al., 2017; Bayliak and Lushchak, 2021; Kostiuchenko et al., 2022), however, data systematization is limited in the case of ischemic stress. Any correction of these signaling molecules’ functioning could be complicated by the variety of regulatory mechanisms involved in the cellular metabolic scenario. A holistic view of mTOR and αKG-mediated interactions may contribute to finding more effective therapeutic strategies for cerebral ischemia. In this mini-review, we discuss the multifunctionality of mTOR and αKG and their collaboration in terms of metabolic homeostasis of neuronal cells under ischemic conditions with a focus on signaling mechanisms involved in endogenous neuroprotection.

## 2. Highlights of mTOR and αKG structure and functions

Mechanistic target of rapamycin is a serine/threonine protein kinase of the phosphatidylinositol-3-kinase-related kinase (PIKK) family that is the basis of two functionally distinct multiprotein complexes: rapamycin-sensitive complex 1 (mTORC1) and relatively rapamycin-insensitive complex 2 (mTORC2). Evolutionarily ancient and highly conserved, TOR is ubiquitously expressed in all eukaryotic cells from yeast to humans, and can be localized both in the nucleus and the cytoplasm (Saxton and Sabatini, 2017; Jhanwar-Uniyal et al., 2019). mTORCs act as convergence centers for incoming environmental stimuli and are important molecular sensors of cellular energy status. The canonical signaling pathway proceeds from extracellular and intracellular stimuli to mTOR via phosphatidylinositol 3-kinase (PI3K), protein kinase B (Akt), tuberous sclerosis complex (TSC), and GTPases, such as Ras homolog enriched in brain/striatum (Rheb/Rhes, respectively) (Bockaert and Marin, 2015). mTORC1 is induced predominantly by fluctuations in amino acids, oxygen, and glucose. At the same time, mTORC2 activity is promoted mainly by growth factors, hormones, and neurotransmitters. mTORs largely coordinate anabolism/catabolism and determine metabolic status and cellular viability (Switon et al., 2017; Schmidt and de Araujo, 2022; Simcox and Lamming, 2022).

Mechanistic target of rapamycin-signaling coordinates basic cell processes such as protein and lipid metabolism, energy homeostasis, mitochondrial and lysosomal biogenesis, proliferation, migration, autophagy, etc. Consistent with its role in coordinating anabolic and catabolic processes, constitutive mTOR activity ensures the adequacy of cellular functioning. In general, mTOR activation is associated with resource/energy sufficiency in the cells (specifically, glucose, oxygen, ATP, amino acids, growth factors, etc.), while mTOR suppression is related to a deficiency of the mentioned factors. Numerous recent studies have significantly deepened the understanding of both upstream mTOR pathways and downstream mTOR targets (Bockaert and Marin, 2015; Castellanos et al., 2016; Saxton and Sabatini, 2017). The main mTORCs-regulated downstream cellular functions are widely described in reviews for different organism systems (Switon et al., 2017; Kostiuchenko et al., 2022). However, many aspects of the molecular interactions that coordinate mTOR-mediated signaling in cerebral physiology and pathology have not yet been fully elucidated. In the brain, mTOR signaling, in addition to basic physiological functions, coordinates specific neuronal functions (e.g., axon regeneration/myelination, dendrite/spine formation, synaptic plasticity, ion channel expression, etc.) underlying cognitive processes (learning, memory, circadian rhythms, etc.) (Bockaert and Marin, 2015; Karalis and Bateup, 2021). Correct modulation of cellular metabolism in response to changing environmental conditions by mTOR signaling is critical to homeostasis.

α-ketoglutarate is a multifunctional molecule that is also actively involved in cellular metabolism as well as maintaining homeostasis in an ever-changing environment. αKG is highly water-soluble, non-toxic, and chemically stable. As a key intermediate in the TCA cycle, it is engaged in the ATP synthesis and balancing the anabolism and catabolism of products/substrates of the TCA cycle (Wu et al., 2016; Kim et al., 2017; Hansen and Gibson, 2022). Endogenous αKG is mainly formed as a result of the oxidative decarboxylation of isocitrate catalyzed by NAD-dependent isocitrate dehydrogenase (IDH). In addition, it is produced through glutaminolysis as a result of glutamate oxidation initiated by transamination catalyzed by an aminotransferase, or oxidative deamination catalyzed by glutamate dehydrogenase (GDH) (Ugur et al., 2017; Andersen et al., 2021; Hinca et al., 2021). Passing further along the TCA cycle, the newly formed αKG increases the probability of ATP generation and thus contributes to the metabolic balance in OGD (Gul and Buyukuysal, 2021).

α-ketoglutarate is primarily present in the mitochondria and cytosol of any cell, besides it is also found in the bloodstream (Wagner et al., 2010). It can cross the blood-brain barrier both by simple diffusion and through a carrier-mediated process (oxoglutarate/malate antiporter, voltage-gated anion channels). This ketoacid exhibits functional features depending on localization, as well as on interactions with other bioactive molecules. Being a cofactor of numerous biochemical reactions in the cell, αKG is involved in multiple physiological processes such as growth, proliferation, neurotransmission, immunity, aging, etc. (Xiao et al., 2016; Zdzisińska et al., 2017; Hansen and Gibson, 2022). αKG contributes to the metabolism of a wide range of bioactive micro and macro molecules (namely, amino acids, proteins, lipids, nucleic acids, carbon, and nitrogen) and thereby modulates many signaling pathways and genes (Yao et al., 2012; Wu et al., 2016).

An important role in the regulation of αKG metabolism and maintenance of cellular homeostasis belongs to the α-ketoglutarate dehydrogenase complex (KGDHC), for which αKG is a substrate. KGDHC is made up of three functionally distinct subunits (E1k, E2k, and E3) that catalyze the conversion of α-ketoglutarate (KG) to succinyl-CoA. The three-step αKG/KGDHC interaction is the rate-limiting step in the TCA cycle. In cooperation with hypoxia-inducible factor 1 (HIF-1) and prolyl-4-hydroxylase (PHD), which controls HIF-1 synthesis/degradation, αKG/KGDHC regulates cellular responses to hypoxia through a feedback loop. In this case, when there is no oxygen present to activate PHD, HIF-1 is stable and activates the E3 ubiquitin-protein ligase SIAH2 which inhibits KGDHC, resulting in the accumulation of αKG that enters the reductive pathway of the TCA cycle and promotes lipid synthesis. αKG produced through glutaminolysis leaves the mitochondria and enters the cytosol leading to the activation of PHD which destabilizes HIF-1 (Kierans and Taylor, 2021; Hansen and Gibson, 2022).

In the brain, in addition to basic functions, αKG determines the balance of neurotransmitter metabolism and neuron-glia communications. This ketoacid can be both a precursor and a metabolite of glutamine and glutamate, according to the cell’s needs (Andersen et al., 2021; Bodineau et al., 2021; Hansen and Gibson, 2022). During recycling between neurons and astroglia, part of the transmitter pool is lost as they are oxidized. The loss can be compensated by their synthesis using αKG as a substrate. Both the excitatory neurotransmitter glutamate and the inhibitory neurotransmitter γ-aminobutyric acid (GABA) can be produced. The increase in the amino acid pool during treatment with exogenous αKG has been shown in experimental models *in vivo* and *in vitro* (Zdzisińska et al., 2017; Bayliak and Lushchak, 2021; Hansen and Gibson, 2022). Although the glutamate-glutamine cycle is balanced under normal homeostatic conditions, αKG is a reliable buffer during critical fluctuations (McKenna et al., 2016; Schousboe, 2019; Rose et al., 2020; Meng et al., 2022). In addition, αKG treatment has been shown to intensify the spatial redistribution of synaptic vesicles, as well as increase the sensitivity of synaptotagmin 1 (Syt1) to Ca^2+^, thereby promoting vesicle fusion with the presynaptic membrane and neurotransmitter release. Along with this, an increase in neurogenesis and an improvement in cognitive functions were observed (Ugur et al., 2017). Thus, in the brain, αKG is a key player in the regulation of both basic neuronal function and the quality/efficiency of synaptic transmission.

## 3. mTOR and αKG signaling in the brain under ischemic conditions

**FIGURE 1:**
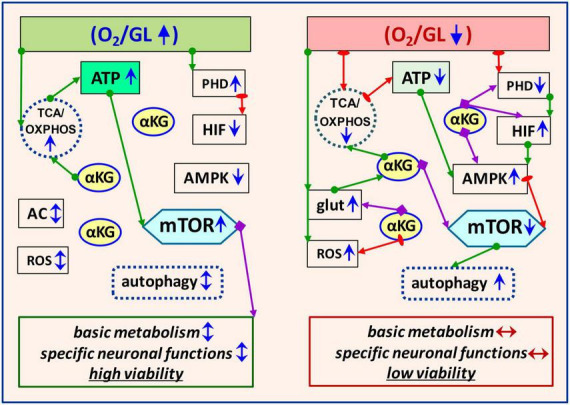
Schematic illustration of key signaling pathways involved in metabolic homeostasis of brain cells in physiological and OGD conditions. The left panel corresponds to normoxia when metabolism is balanced to ensure adequate brain cell functions. The right panel shows the OGD-induced mechanisms involved in equilibrating the metabolic imbalance. Modulatory effects of the molecules are marked with purple arrows, activation–with green arrows, and inhibitory effects–with red. Short arrows near the molecule or process indicate its functional activity (blue sharp–high or low, blue/red double-edged–balance/imbalance) under normoxia or OGD. GL, glucose; TCA, tricarboxylic acid cycle; OXPHOS, oxidative phosphorylation; AC, amino acids; glut, glutamate; AMPK, 5′AMP-activated protein kinase; PHD, oxygen-sensing prolyl hydroxylase domain enzymes; HIF, hypoxia-inducible factor; ROS, reactive oxygen species.

The cellular response to OGD is initiated primarily through oxygen- and AMP/ATP-sensitive molecules-sensors–HIF-1, and 5′AMP-activated protein kinase (AMPK), respectively. In close cooperation with mTOR/αKG-mediated mechanisms, these molecules are responsible for an adequate cellular response to OGD. In normoxia, when O_2_ and AMP/ATP are balanced (Figure 1), HIF-1 and AMPK are predominantly inactive. In brain hypoxia/ischemia, PHD is downregulated, followed by upregulation of HIF-1 expression and HIF-mediated induction of transcription of multiple target genes to rearrange metabolism for adaptation. In parallel, AMPK is activated as a result of an increase in the AMP/ATP and ADP/ATP ratios, indicating an energy deficit caused by ischemia. Moreover, HIF-1 can also upregulate AMPK through REDD1 (REDD1–regulated DNA damage and development 1 protein). Activation of both mentioned sensor molecules (HIF and AMPK) triggers compensatory mechanisms programming brain cells to provide homeostasis and/or recovery processes.

Many data indicate that ischemia-induced neurodegenerative processes are associated with the dysregulation of mTOR- and αKG-signaling involving AMPK and HIF-1 intermediates (Stegen et al., 2016; Saxton and Sabatini, 2017; Kierans and Taylor, 2021; Li et al., 2021; Villa-González et al., 2022). OGD induces mTOR suppression and αKG accumulation, followed by an increase in autophagy and compensatory activation of ATP production due to the incorporation of excess αKG into the TCA cycle (Hansen and Gibson, 2022; Villa-González et al., 2022). It is known that under normoxic conditions, mTOR mainly blocks autophagy, but does not exclude it. The functional relationship between the mTOR pathway and autophagy involves complex regulatory signaling loops aimed at maintaining metabolic balance (Deleyto-Seldas and Efeyan, 2021). It has been shown that autophagy activation may result not only from AMPK-mediated mTOR suppression but also from direct phosphorylation of ULK1, a serine/threonine protein kinase involved in autophagy (Mo et al., 2020). At a certain stage, these processes are adaptive-protective, however, excessive autophagy can exacerbate brain damage. Treatment of animals with the mTOR inhibitor rapamycin after ischemia has shown neuroprotective effects and autophagy activation, but studies of its effects in other models of cerebral ischemia/reperfusion describe conflicting results (Beard et al., 2019). In the case of hyperactivation, the autophagy inhibitor LiCl activated mTOR signaling, promoting repair processes and improving neuronal viability (Hadley et al., 2019; Hou et al., 2019; Zhang et al., 2021). Thus, in the context of metabolic homeostasis, mTOR activation or suppression is appropriate at different stages of OGD. Besides, let us note that the effects of ischemia are determined both by the intensity and duration of ischemia/reperfusion and cell type, as well as by individual cell specificity and resistance. For example, in a focal ischemia model, mTORC1 is downregulated in the ischemic nucleus, but mTORC1 is upregulated in the ischemic penumbra (Wu et al., 2017; Villa-González et al., 2022). mTOR signaling dynamics vary widely across neuronal and glial cell types (McKenna et al., 2016; Cespedes et al., 2019; Schousboe, 2019; Rose et al., 2020). To maintain cellular homeostasis under OGD conditions, coordination of functional interactions of HIF/AMPK/mTOR/αKG molecules is required. Such signaling cooperation manifests itself according to the physiological expediency for a particular cell type under specific OGD conditions. It should be noted that the mobility and versatility of αKG allow it to provide metabolic plasticity at the cellular level.

**FIGURE 2:**
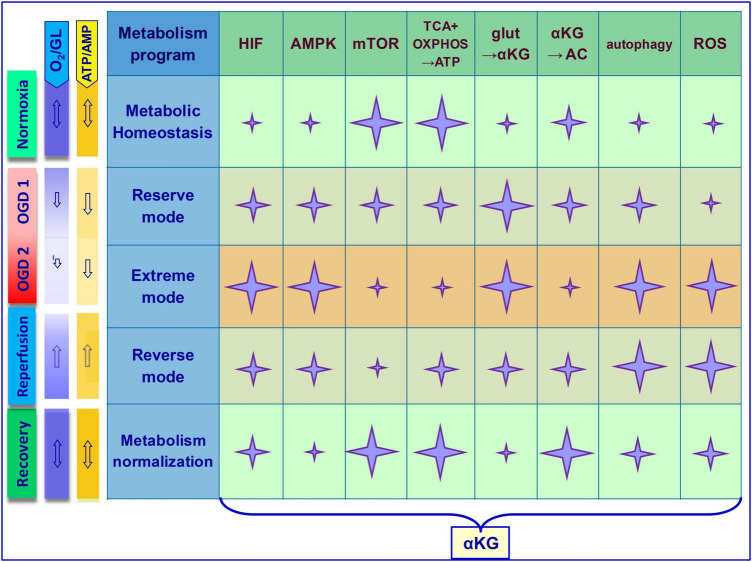
Multifunctional molecules in the metabolic dynamics of brain cells under ischemic exposure and recovery of homeostasis during reperfusion. The scheme shows key OGD-induced cellular events. Stages–Normoxia, OGD 1 (O_2_, ATP–reduced), OGD 2 (O_2_, ATP–depleted), reperfusion, recovery. Metabolism programs–Metabolic homeostasis, reserve mode, extreme mode, reverse mode, metabolism normalization. Involvement of basic functional molecules and mechanisms in the cellular response to OGD (marked with a star). Star size correlates with activity level. Note that at different stages, αKG can directly or indirectly contribute to the equalization of metabolic homeostasis through opposite mechanisms. In OGD, αKG promotes ATP synthesis, glutamate metabolism (glut→αKG), mTOR suppression, autophagy activation, and ROS neutralization. During the recovery stage after OGD, αKG promotes additional ATP synthesis, amino acid metabolism (αKG→AC), mTOR activation, and normalization of autophagy and ROS. Correspondence of arrows (double-edged–balance, sharp–decrease or increase in the indicator). GL, glucose; TCA, tricarboxylic acid cycle; OXPHOS, oxidative phosphorylation; AC, amino acids; glut, glutamate; AMPK, 5′AMP-activated protein kinase; PHD, oxygen-sensing prolyl hydroxylase domain enzymes; HIF, hypoxia-inducible factor; ROS, reactive oxygen species.

Although the protective properties of exogenous αKG have been widely described in the treatment of brain diseases including cerebral ischemia (Zdzisińska et al., 2017; Meng et al., 2022), the molecular bases of these effects are still being studied. Stabilization of metabolic homeostasis in OGD involving mTOR/αKG signaling can be achieved by multidirectional events that become relevant at different stages during/after ischemia. Generally, metabolic transformations are oriented on the balance of ATP, glutamate, mTOR, and ROS in brain cells. In experimental ischemia models *in vivo* and *in vitro*, it was noted that conversion of glutamate into αKG can contribute to both the activation of ATP production and the reduction of glutamate excitotoxic effect, preserving viability and function of neurons in OGD (Kim et al., 2017; Ferrari et al., 2018; Andersen et al., 2021; Hinca et al., 2021). In addition, αKG has been shown to promote the synthesis of the amino acid L-carnitine, which is critical for the efficient metabolism of fatty acids into ATP (Thomas et al., 2015). These events are most relevant during and immediately after the OGD, which corresponds to OGD1 in Figure 2.

In the context of αKG/mTOR cooperation to maintain homeostasis under OGD conditions, many studies have shown that αKG indirectly inhibits mTOR with subsequent activation of autophagy. The effects of αKG may be mediated by the activation of HIF-1 and AMPK, as well as the inhibition of ATP synthase, which is a direct target of αKG (Chin et al., 2014; Su et al., 2019; Kostiuchenko et al., 2022). This may be an additional or alternative mechanism for the downregulation of mTOR functions by αKG that promotes cell survival in OGD. Such a scenario is most appropriate in the subsequent ischemic period, which corresponds to OGD2 in Figure 2.

When cells acquire resilience as a result of oxygen and glucose stabilization (Reperfusion) and ATP saturation in the environment, mTOR activation becomes relevant, which can also be mediated by αKG via HIF-1 and AMPK downregulation. αKG is a cofactor of many oxygenases that provide oxidation reactions involving molecular oxygen and determine oxygen homeostasis in cells (Hansen and Gibson, 2022). In particular, αKG activates PHD with subsequent suppression of PHD-dependent HIF signaling, thereby indirectly stabilizing the effects of HIF-1 (Stegen et al., 2016; Kierans and Taylor, 2021; Li et al., 2021). It should be noted that this variant of the event, taking into account the αKG excess in the cytoplasm, suggests the possibility of a metabolic shift toward the activation of the mTOR signaling pathway even under OGD conditions. In the post-ischemic period, mTOR-mediated synthetic processes are of great importance for ensuring homeostasis and restoring neuronal functions. There are results indicating activation of the mTOR signaling pathway by αKG as a biosynthetic precursor for amino acids such as glutamate, glutamine, leucine, and proline. αKG treatment increases the levels of phosphorylation of mTOR, 4E-BP1, and p70 S6K1, thereby promoting the initiation of protein synthesis. In addition, glutamine-derived αKG has been reported to activate mTORC1 via GDP/GTP-mediated signaling (Yao et al., 2012; Simcox and Lamming, 2022).

Moreover, αKG has been found to inhibit the inflammatory response and oxidative damage in the brain during ischemic injury. The authors point out that these effects may be mediated by the c-Fos/IL-10/stat3 signaling pathway (Hua et al., 2022). Thus, the antioxidant properties of αKG also may explain its protective effects. It contributes to ROS scavenging, as it can directly neutralize hydrogen peroxide, superoxide, and other reactive species, as well as increase the enzymatic activity of superoxide dismutase, catalase, and glutathione peroxidase (Liu et al., 2018).

Multiple neurodegenerative diseases accompanied by ischemic phenomena are associated with a decrease in KGDHC activity and accumulation of αKG, which may contribute to the implementation of αKG multifunctionality (Johnson et al., 2020; Gul and Buyukuysal, 2021; Hansen and Gibson, 2022; Meng et al., 2022). Thus, αKG is critical for metabolic plasticity, homeostasis, and viability of brain cells during ischemia through its involvement in the regulation of key metabolic processes (namely, energy production, amino acid metabolism, modulation of signaling pathways, and antioxidant defense).

## 4. Discussion

The status of brain cell homeostasis has been shaped evolutionally. Although the structure of biologically active molecules is conserved, their functional efficiency in cellular metabolism is determined by the plasticity of intermolecular communications. The involvement of numerous intermediates in ischemia-induced signaling processes can make cellular responses ambiguous, and the consequences for cells and the organism as a whole can be uncontrollable. However, the inherent cellular programs of self-regulation can maintain metabolic homeostasis within certain limits of OGD fluctuations. Neuronal metabolism is most sensitive to OGD compared to more resistant glial cells. In cerebral ischemia, the degree of OGD and individual metabolic programs of neuronal and/or glial cells determine the triggering of efficient molecular events that determine tissue homeostasis. mTOR and αKG signaling substantially mediate these processes, which manifest themselves according to the specificity of metabolism in various cell types. Under conditions of transient and reversible ischemia, metabolic dynamics can hypothetically be divided into several stages and characterized in the context of the mentioned signaling molecules. Namely, normoxia corresponds to metabolic homeostasis, OGD begins with a “reserve mode” using internal resources (OGD1), moving on to an “extreme mode” (OGD2) in case of energy depletion. It is assumed that when the disturbances can still be reversible, autophagy is activated, but cell damage/death becomes inevitable when the homeostatic threshold is exceeded. Oxygen-glucose stabilization of the extracellular environment (otherwise, reperfusion) corresponds to the “reverse mode” followed by the “metabolism normalization.” The mechanistic model of metabolic dynamics in OGD and the involvement of the key molecules considered in this mini-review are presented in Figure 2.

The coordinated interaction of molecules, including mTOR and αKG, is of great importance for ensuring the gradual transition of cellular metabolism from a normal state to an extreme one, and then to a reverse one, followed by a return to metabolic homeostasis when the environment stabilizes.

Based on the available data, we hypothesize that the maintenance of metabolic homeostasis involves the support of ATP synthesis, the suspension of basic normoxic programs (anabolism/catabolism, etc.), the removal of critical consequences (glutamate metabolism, activation of autophagy, and ROS scavenging), the activation of stabilization/recovery programs due to existing cellular resources and expediency. In the context of ischemia-induced events, the multifunctional molecules mTOR and αKG can be mechanically defined as cellular “integrator” and “equalizer,” respectively, which implement programs of endogenous neuroprotection. Taking into account the multifunctionality and mobility of αKG, its abundance should increase the survival of brain cells in OGD. An integrated approach to understanding ischemia-induced molecular mechanisms of regulation of the mTOR signaling pathway, including the involvement of αKG, can increase the effectiveness of exogenous neuroprotection in ischemia-induced disorders.

## Author contributions

IL: conceptualization and drafting the manuscript. OK: literature analysis and revising the manuscript. MK: reviewing the manuscript. GS: supervision and reviewing the manuscript. All authors read and agreed to the published version of the manuscript.
